# Green Fiber-Reinforced Laminates: Styrene-Free UPe with VTES-Functionalized ZrO_2_ and Flax Fabrics

**DOI:** 10.3390/polym18010070

**Published:** 2025-12-26

**Authors:** Slavko Mijatov, Milica Rančić, Tihomir Kovačević, Jelena Vujančević, Vladimir B. Pavlović, Jelena D. Gržetić

**Affiliations:** 1 Military Technical Institute, Ratka Resanovića 1, 11030 Belgrade, Serbia; slavko.mijatov@mod.gov.rs (S.M.); tkovacevic@tmf.bg.ac.rs (T.K.); 2 Faculty of Forestry, University of Belgrade, Kneza Višeslava 1, 11030 Belgrade, Serbia; milica.rancic@sfb.bg.ac.rs; 3 Institute of Technical Sciences of SASA, Knez Mihailova 35/IV, 11000 Belgrade, Serbia; jelena.vujancevic@itn.sanu.ac.rs; 4 Faculty of Agriculture, University of Belgrade, Nemanjina 6, Zemun, 11080 Belgrade, Serbia; vladimirbpavlovic@gmail.com

**Keywords:** flax, fiber-based composites, styrene-free UPe, zirconia nanoparticles

## Abstract

Natural fiber-based composites are gaining attention as sustainable alternatives to synthetic fiber-reinforced materials. Herein, styrene-free unsaturated polyester (UPe) nanocomposites and flax-fabric laminates reinforced with vinyl-triethoxy-silane (VTES) functionalized zirconia nanoparticles (ZrO_2_-VS) were studied. Nanoparticles were dispersed by high-shear mixing, and ZrO_2_-VS was benchmarked against unmodified ZrO_2_ and neat UPe. Fourier-transform infrared spectroscopy (FTIR) tracked cure conversion; scanning electron microscopy (SEM/EDS), tensile testing, and dynamic mechanical analysis (DMA) evaluated structure-property relationships. ZrO_2_-VS improved dispersion and interfacial adhesion, yielding higher tensile strength and storage modulus compared with unmodified ZrO_2_. In flax-fabric laminates, ZrO_2_-VS/UPe achieved a tensile strength of 72.2 ± 3.6 MPa, exceeding both unmodified ZrO_2_/UPe and neat UPe controls. DMA showed pronounced increases in storage modulus across temperature with small, non-significant changes in Tg. These results highlight a low-styrene-hazard UPe matrix and natural fiber reinforcement pathway to improved mechanical performance via silane-mediated nanoparticle-matrix-fiber bridging.

## 1. Introduction

The development of natural fiber–reinforced polymer composites (NFRPCs), as environmentally sustainable materials, has attracted significant attention due to growing concerns over the environmental, economic, and health impacts of synthetic fibers. These impacts originate from the use of non-renewable raw materials, high carbon emissions, significant energy consumption, and the non-renewable and non-biodegradable nature of synthetic fibers [1,2]. In contrast, natural fibers offer advantages such as low density, high availability, renewability, recyclability, low cost, and competitive mechanical strength, making them promising eco-friendly alternatives for reinforcing polymer composites [2]. As a result, increasing environmental awareness, energy challenges, and the need to reduce dependence on petroleum-based products have driven researchers to explore natural fibers as viable replacements for traditional synthetic reinforcements.

Plant-based natural fibers such as flax, ramie, hemp, and roselle have been widely used as reinforcements in polymer matrices in various forms—particle, fiber, or structural laminates [3,4,5,6]. Laminated composites are particularly advantageous when mechanical performance is prioritized, offering high specific strength and stiffness [7], along with excellent fatigue and corrosion resistance. Among those, flax fibers have gained significant attention due to their excellent tensile properties, low density, biodegradability, and high cellulose content, making them one of the most promising reinforcements for many structural and semi-structural applications [8,9,10,11]. In addition, flax exhibits good adhesion with a variety of polymer matrices and has been extensively studied in hybrid and laminated composite forms. Recent advancements have shown that composites reinforced with treated flax fibers can achieve mechanical properties comparable to those reinforced with glass fibers [9]. For example, supercritical CO_2_ treatment was demonstrated to enhance the modulus and strength of flax fibers by up to 40%, while reducing porosity and improving fiber-matrix bonding. Applications of flax fiber composites include automotive, aerospace, construction, and even biomedical fields due to their favorable strength-to-weight ratio, recyclability, and reduced environmental footprint [10].

The mechanical and dynamic-mechanical properties of NFRPCs depend on fiber properties (aspect ratio (length/diameter), fiber weight and volume fractions) as well as polymer matrix properties [12,13]. Hence, selecting a compatible polymer matrix is crucial for ensuring strong interfacial bonding. Unsaturated polyester (UPe) resins are widely used as low-cost thermosetting matrices due to their room-temperature processability and excellent mechanical properties. Styrene is commonly employed as a reactive diluent in UPe formulations to reduce viscosity and facilitate fiber wetting, while also contributing to crosslinking [14,15,16]. However, as a volatile organic compound (VOC), styrene possesses health and environmental hazards, prompting the development of styrene-free alternatives [16,17].

One of the key challenges in NFRPCs is the weak interfacial interaction between hydrophilic natural fibers and hydrophobic polymer matrices. Although styrene-free UPe systems offer safer alternatives, achieving effective fiber wetting and bonding remains a scientific and technological challenge [16]. To address this, various approaches have been explored, including the surface modification of natural fibers using organic silanes. Using vinyltriethoxysilane (VTES) functionalization of natural fibers provides higher cross-linking reactivity during the curing of the composite laminate, better compatibility, and physical reinforcing via achieving two main types of intermolecular interactions: π,π-stacking, and hydrogen bonding interactions [18].

Furthermore, the incorporation of inorganic fillers enhances composite performance by improving strength and modulus, achieving properties comparable to metallic materials used in structural applications [19]. Numerous studies have demonstrated that nano- and micro-sized metal, metal oxide, and ceramic particles can significantly enhance the physical, mechanical, and thermal properties of thermosetting polymer composites [20]. Rigid metal oxide fillers such as CaCO_3_, CaO, MgCO_3_, and MgO have been reported to increase hardness and water resistance in UPe composites. Similarly, hybrid jute/glass composites exhibited higher ultimate tensile strength than composites reinforced solely with jute [21]. Moreover, UPe composites fabricated via hand lay-up with jute fiber and Al_2_O_3_ or ZrO_2_ particles showed enhanced thermal stability and lower moisture absorption, making them suitable for structural applications. Silica was also used effectively as a filler due to its high surface area, low cost, and ease of surface modification [4]. Zirconia nanoparticles are increasingly used as inorganic reinforcements in polymer matrices due to their high stiffness, hardness and thermal stability, which can translate into improved flexural strength, fracture toughness and surface hardness of the host polymer [22]. In fiber-reinforced systems, hydrous ZrO_2_ nanoparticles grafted onto flax fibres have been shown to significantly increase tensile and interfacial bonding strength, demonstrating their potential as efficient interfacial modifiers in natural fibre composites [23].

In light of the need for sustainable and high-performance engineering materials, this study focuses on the development of eco-friendly composite laminates based on styrene-free UPe matrix and woven flax fibers (flax fabric). Zirconium oxide nanoparticles (n-ZrO_2_), previously functionalized/coated using VTES (ZrO_2_VS), were integrated as an organometallic coupling agent into the UPe matrix to improve compatibility and interfacial bonding of UPe and flax fabric.

## 2. Materials and Methods

### 2.1. Materials

Silane coupling agents (VTES, vinyltriethoxysilane) were supplied by Sigma Aldrich (Merck KGaA, Darmstadt, Germany). The ZrO_2_ (99%, 40–50 nm) nanoparticles were purchased from Nanografi Advanced Materials(Ankara, Turkey). Ethanol absolute p.a. was supplied by Zorka Pharma-Hemija Ltd (Šabac, Serbia). Styrene-free unsaturated polyester (UPe, commercial name Yachtcare Azur VT) and methyl ethyl ketone peroxide (commercial name MEKP FL 505 SN) were supplied by Vosschemie, Uetersen, Germany. Yachtcare Azur VT is a cobalt-accelerated thixotropic polyester resin (specific gravity 1.1 g·cm^−3^) based on orthophthalic acid and maleic anhydride. Vinyl toluene (mixture of m-methylstyrene and p-methylstyrene) was used as UPe resin solvent due to its lower toxicity and volatility compared to styrene. FlaxdryTM BL200 (woven flax fiber fabric) was supplied by EcoTechnilin, Valliquerville, France. Flax fabric properties are shown in Table 1.

### 2.2. Preparation of the VTES-Coated ZrO2 Nanoparticles

For the hydrolysis reaction, a 5 vol.% solution of the VTES coupling agent was prepared using an ethanol/water mixture (ethanol/water/VTES = 45 cm^−3^/2.5 cm^−3^/2.5 cm^−3^) as described by Song et al. [28]. The solution was vigorously stirred at room temperature in a glass three-necked round-bottom flask, equipped with a condenser and magnetic stirrer to initiate hydrolysis. Subsequently, 2.0 g of ZrO_2_ nanoparticles were added to the mixture and stirred at 100 °C for 3 h to facilitate surface modification. The VTES-coated ZrO_2_ (ZrO_2_VS) nanoparticles were then dried under vacuum at 100 °C for 24 h to promote condensation reactions and complete the silane grafting process [29]. A schematic illustration of the ZrO_2_ nanoparticle surface treatment process is provided in Figure S1.

### 2.3. Preparation of Nanocomposites Based on Styrene-Free Polyester Resin and VTES-Coated ZrO_2_ by Solution Dispersion Method (Blending Method)

**Figure 1:**
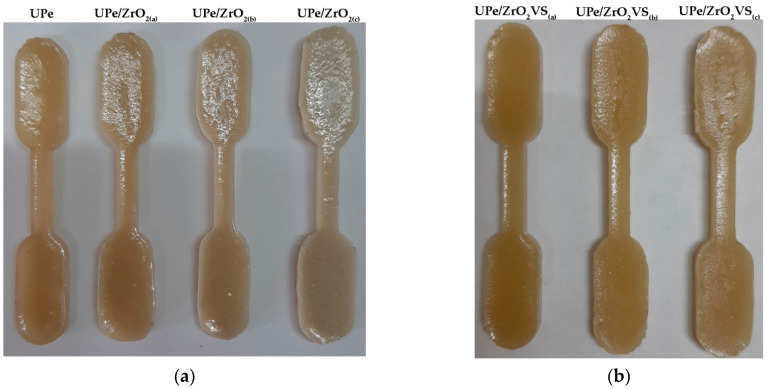
Standard samples of (**a**) cured UPe resin and (**b**) composites based on UPe resin and different ratios of unmodified ZrO_2_ and coated ZrO_2_VS nanoparticles.

Nanocomposites based on styrene-free unsaturated polyester (UPe) and either unmodified or VTES-coated ZrO_2_ nanoparticles were prepared by the solution blending method [18]. Unmodified ZrO_2_ or VTES-coated ZrO_2_ (ZrO_2_VS) nanoparticles were previously dispersed in UPe matrix for 10 min using an ultrasonic bath (Bandelin electronic, Berlin, Germany, power 120 W, frequency 35 kHz), maintaining the temperature at 23 °C. Afterwards, the 1.5 wt.% MEKP catalyst was added, and the mixture was dispersed for an additional 5 min and transferred to a polytetrafluoroethylene (PTFE) mold to obtain standard rectangular or dog-bone (Figure 1) samples for mechanical testing. Ultrasonic nanoparticle dispersion was applied to ensure uniform distribution within the UPe matrix [18]. Samples were cured for 24 h at 25 °C, and then post-cured at 80 °C in a laboratory oven for 6 h [30]. The resulting nanocomposites were designated as UPe/R_(n)_, where R indicates the type of nanoparticle (ZrO_2_ for unmodified and ZrO_2_VS for VTES-modified), and n represents the ZrO_2_ nanoparticle loading: 1.5 wt.% (a), 3.0 wt.% (b), and 5.0 wt.% (c).

### 2.4. Preparation of the Multi-Layer Composite Laminate by Combined Hand Lay-Up and Vacuum-Assisted Molding Technique (VARTM)

**Figure 2:**
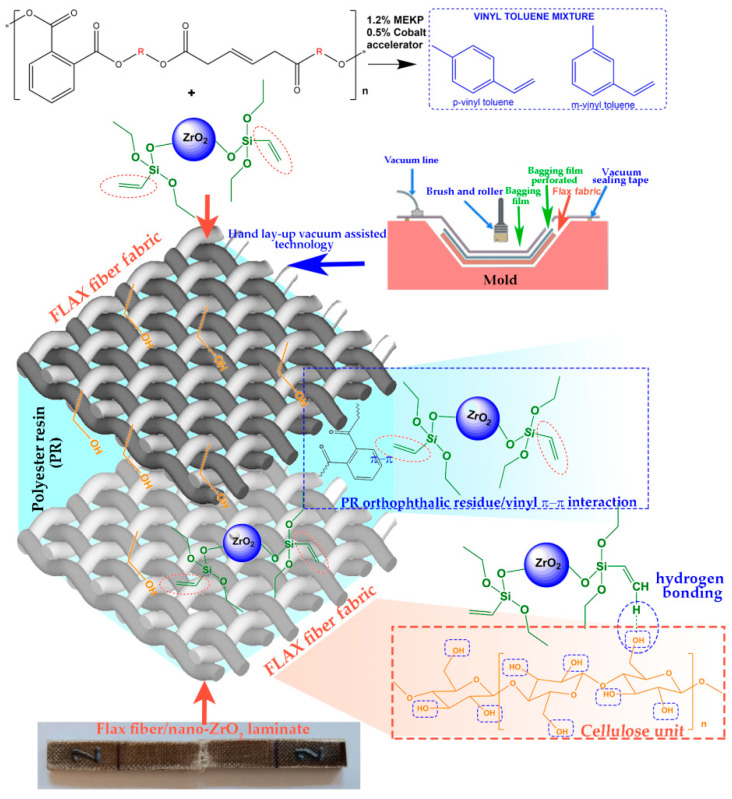
Schematic illustration of the reinforcing interactions between UPe, VTES-coated ZrO_2_ nanoparticles, and flax fabric.

For mechanical properties evaluation, composite laminates with dimensions of 250 mm × 270 mm with a target flax fabric-to-resin weight ratio of 60:40 were fabricated using a combined hand lay-up and vacuum-assisted molding technique [30], from which individual specimens were subsequently cut and lightly trimmed to the required dimensions to ensure uniform geometry. Initially, 5 wt.% of either unmodified ZrO_2_ or coated ZrO_2_VS nanoparticles were homogenized in the UPe resin for 10 min using the ultrasonic bath (Bandelin electronic, Berlin, Germany, power 120 W, frequency 35 kHz), maintaining the temperature at 23 °C, and then 1.5 wt.%. MEKP catalyst was added and homogenized for an additional 5 min. The resulting nanoparticle dispersions were manually applied by brush and roller onto flax fabric sheets (layers) of dimensions adapted to the PTFE molds, which were used for laminate preparation (250 × 270 mm). The flax fabric-to-resin weight ratio was maintained at 60:40. To ensure proper soaking of flax fabric by resin, the 2 mbar vacuum was applied, whereas UPe excess was removed through perforated bagging film—Figure 2. The applied vacuum is 99.8% below standard atmospheric pressure (~1013 mbar), and it minimizes void formation within the initial laminate curing at ambient temperature (25 °C) for 24 h. Samples were post-cured at 80 °C for 6 h. After curing, excess resin was mechanically removed using a lathe to achieve uniformity. This post-processing step did not disturb the fiber orientation and thus had no adverse effect on the mechanical properties of the composite. The final plate thickness, determined by the PTFE mold dimensions, was 5 mm, thereby attaining the volume-to-fraction ratio (*V_f_*) of 0.708.

Given the demand for sustainable and high-performance materials, this study aims to develop environmentally friendly composite laminates based on styrene-free UPe matrix and flax fabric. To enhance compatibility and interfacial adhesion between the non-polar UPe matrix and polar flax fabric, the UPe matrix was pre-filled using 5 wt.% unmodified ZrO_2_ or ZrO_2_VS coated nanoparticles. Mijatov et al. [30] performed theoretical calculations to predict intermolecular forces that occur in UPe/flax fabric/kraft lignin composite laminate. Authors found that hydrogen bonding was significant for the flax fiber/UPe interactions, which involve interactions between the free carboxylate group from the maleic acid segment of the UPe structure and hydroxyl groups of flax fiber [30]. A schematic illustration of the intermolecular interactions in the composite laminate is presented in Figure 2.

### 2.5. Characterization Methods

#### 2.5.1. FTIR Analysis

Fourier transform infrared spectroscopy (FTIR) spectra of the raw materials (ZrO_2_ and ZrO_2_VS) and cured composite were recorded in transmittance mode using a Nicolet™ iS™ 10 FTIR Spectrometer (Thermo Fisher Scientific, Waltham, MA, USA) with Smart iTR™ FTIR sampling accessories, within a range of 400–4000 cm^−1^, at a resolution of 4 cm^−1^ and in 20 scan mode.

#### 2.5.2. Particle Size Determination

Particle size distribution and average particle size of unmodified ZrO_2_ particles were determined utilizing a Particle Size Analyzer (PSA 1190 L/D, Anton Paar, Graz, Austria ) in liquid operation mode by dispersing the powders using an ultrasonic unit within the device.

#### 2.5.3. SEM/EDS Analysis

Furthermore, the morphology, size, and shape of ZrO_2_ and ZrO_2_VS, as well as flax fabric structural analysis and failure analysis of the cured composites and samples after mechanical characterization, were investigated using scanning electron microscopy (SEM) and energy dispersive X-ray technique (EDS). Samples were first coated with gold for 100 s at 30 mA, on a Baletic device SCD 005 Sputter Coated. The SEM micrographs were recorded using a JEOL JSM-639LV SEM microscope (JEOL Ltd., Akishima, Tokyo, Japan) coupled with an electron dispersive spectroscope (EDS, Oxford Instruments, X-MaxN, High Wycombe, UK).

#### 2.5.4. Mechanical Characteristics

Uniaxial tensile measurements of standard cured samples, followed ASTM D882 [31] (Standard test method for tensile properties of thin plastic sheeting, 2009), were performed using an Instron 1122 tester. All tests were performed at 20 °C, adjusted at a crosshead speed of 1 mm‧min^−1^. The five samples were prepared and were tempered in the temperature chamber at 20 °C for two h. The fracture surface after the test was inspected and standard deviation was calculated for regular samples.

#### 2.5.5. DMA Analysis

Dynamic mechanical analysis (DMA) of cured composites was performed using the Modular Compact Rheometer MCR–302 (Anton Paar GmbH) in torsion deformation mode. MCR–302 was equipped with standard fixtures for rectangular bars (SRF12) and a high-temperature stability chamber (±0.1 °C CTD–620). The standard sample of a rectangular bar shape (44 × 10 × 4 mm) was tested by using a “temperature ramp test” at a temperature range from 40 °C to 160 °C. The heating rate was 5 °C ·min^−1^, the single angular frequency 1 Hz, and the strain amplitude 0.1%.

Reinforcement efficiency factor for developed UPe/R(n) nanocomposites was calculated according to the Einstein Equation (1) from the “temperature ramp test” results [32]:

G’_c_GS50 °C_ = G’_UPe_GS50 °C_⸱(1 + rV_f_) (1)

where G’_c_GS50 °C_ and G’_UPe_GS50 °C_ are the storage modulus of the composite and UPe matrix at the glassy state, respectively. V_f_ is the volume fraction of the ZrO_2_ and ZrO_2_VS filler, and r is the reinforcement efficiency factor.

## 3. Results and Discussion

### 3.1. FTIR Analysis and Curing Kinetics

Fourier-transform infrared spectroscopy (FTIR) was used to (i) verify the presence of VTES-derived linkages on ZrO_2_ (ZrO_2_VS) and their retention in the laminates and (ii) quantify cure conversion of the styrene-free UPe matrix and UPe/ZrO_2_ nanocomposites.

#### 3.1.1. FTIR Spectra of Raw Materials and Nanocomposites

**Figure 3:**
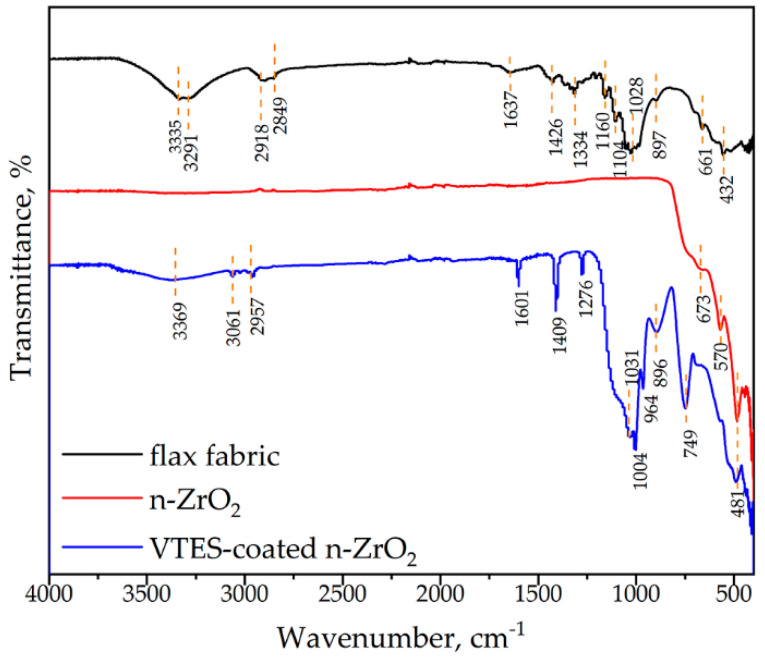
FTIR spectra of the raw materials: flax fabric, unmodified ZrO_2,_ and VTES-coated ZrO_2_ nanoparticles.

**Figure 4:**
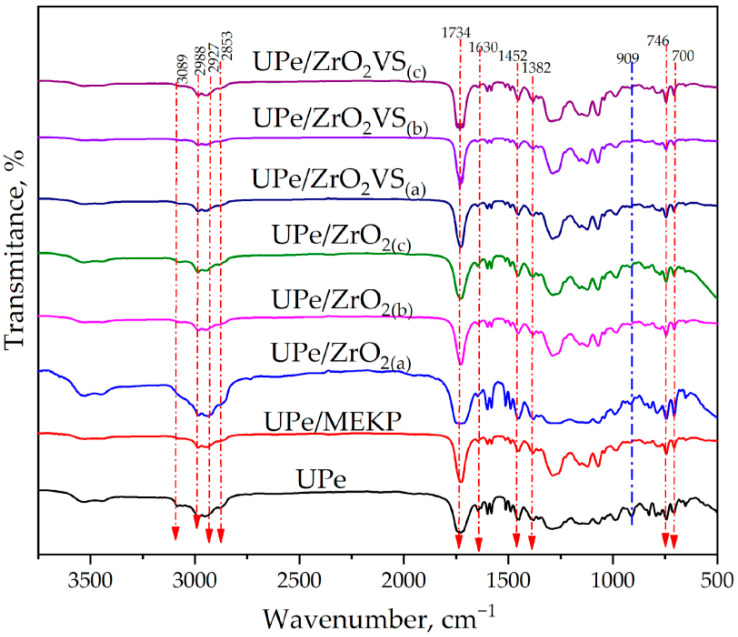
FTIR spectra of the UPe/R(n) nanocomposites.

The FTIR spectra display the vibrational characteristics of multiple samples, including raw flax fiber, nano-ZrO_2_ (modified/unmodified) (Figure 3), styrene-free UPe resin, and corresponding composites (Figure 4). The FTIR of the raw flax fabric showed the adsorption bands in the region 3335–3291 cm^−1^ due to stretching vibration of the surface hydroxyl groups from cellulose, hemicellulose, and absorbed moisture—characteristic of natural fibers. The bends observed around 2918 cm^−1^ and 2849 cm^−1^ originate from asymmetric and symmetric stretching vibration of the C-H in methyl and methylene groups from organic components of flax. They are consistent across all samples, confirming the presence of the polymer backbone. Moreover, an adsorption band at 1637 cm^−1^ is observed due to carbonyl C=O vibration (Figure 3), while C=C stretching peaks were observed in the range of 1483–1590 cm^−1^. Peaks between 500–800 cm^−1^ are associated with metal-oxygen (Zr–O) stretching vibrations, confirming the presence of ZrO_2_ nanoparticles in the modified particles or composites. Variations in this region between modified and unmodified ZrO_2_ can indicate successful surface functionalization (e.g., silane coating).

The observed FTIR peaks in the 498–502 cm^−1^ for the unmodified ZrO_2_ and ZrO_2_VS nanoparticles are attributed to the vibration modes of ZrO_3_^2−^ groups [33]. A Si–C stretching peak was observed at 749 cm^−1^ for ZrO_2_VS. The broad absorption peak of silanol hydroxyl group (Si–OH), observed around 3369 cm^−1^, originate from hydrolysis of Si–O–C group within VTES [34]. A shoulder peak at 1045 cm^−1^ was attributed to the overlapped Si–O–C stretching vibrations. Compared with pristine ZrO_2_, ZrO_2_VS exhibits new/strengthened bands attributable to silane grafting (Figure 3): (i) Si–O–Si/Si–O–C stretching in the 1130–1030 cm^−1^ envelope; (ii) C–H wagging/rocking features from the vinyl-bearing organosilane at ~1400–900 cm^−1^; and (iii) a shoulder consistent with Zr–O–Si linkages in the ~950–980 cm^−1^ region. The relative integrated intensity of the 1130–1030 cm^−1^ envelope increases upon silanization. Residual ethoxy groups (Si–O–C_2_H_5_) are minimal after curing/solvent washing, as suggested by the weak/absent ~2970–2870 cm^−1^ alkyl stretches. These results implied that the VTES coating on the surface of ZrO_2_ particles was successfully achieved.

The FTIR spectra of (UPe) resin and corresponding composite formulations (Figure 4) showed a strong, sharp peak at ~1730 cm^−1^ (C=O stretching) associated with ester carbonyl groups in the unsaturated polyester matrix. The intensity of this peak slightly decreases in composites, depending on the interaction between the matrix and fillers or fibers. Bands around 1250–1050 cm^−1^ (C–O stretching) are typical of ester linkages in the UPe resin. Shifts or intensity changes in this region can suggest interactions between the matrix and added fillers (e.g., bonding with modified ZrO_2_ or flax hydroxyl groups). In fracture-surface ATR spectra of UPe/ZrO_2_VS/flax laminates, the Si–O–Si envelope persists (Figure 4), indicating interphase retention through cure.

#### 3.1.2. FTIR Analysis of Curing Kinetics

FTIR was used to track curing kinetics and evaluate how VTES functionalization of ZrO_2_ nanoparticles affects crosslinking of the styrene-free UPe matrix in the composite. Because resin processability and cure behavior control laminate quality in hand lay-up/vacuum-assisted processing—especially when the resin is pre-filled with reactive fillers—the aim is to balance flow and gelation, remove excess resin under vacuum, and achieve full cure for the desired properties. Minimization of curing rate and an adequate removal of excess resin, together with ensuring complete curing to achieve desired mechanical properties, is the primary goal during the laminate production. Cure was monitored via the decrease of the height of the vinyl toluene C=C out-of-plane deformation band at ~909 cm^−1^ (marked blue on Figure 4), which participates in the crosslinking reaction [35]. The degree of conversion, α(t), was calculated from the normalized 909 cm^−1^ (Figure S3a) band height and reference ester C=O peak around 1734 cm^−1^ (Figure S3b) using Equation S1 and plotted versus time (Figure S4). The times to reach 50% and 90% conversion (t_50_, t_90_) and the final conversion (α_final_) are summarized in Table 2.

As shown in Figure S3 and Table 2, both nanoparticle loading and surface functionalization markedly influence the UPe cure. FTIR cure tracking shows stage-dependent effects of nanoparticles: unmodified ZrO_2_ perturbs early/late stages differently, whereas ZrO_2_VS maintains or improves conversion at late times. Thus, silanization enhances compatibility without blocking network build-up.

For unmodified ZrO_2_, the effect depends on the stage of reaction [36]. In the early period, quantified by the time to 50% conversion (t_50_), the slowest cure occurs for UPe/ZrO_2_(a) (1.5 wt% ZrO_2_), with t_50_ = 25 min. Increasing the loading of unmodified ZrO_2_ lowers t_50_, indicating faster early conversion, but this comes at the expense of the final conversion (α_final_), which drops in the highest-loaded sample. These trends are consistent with progressive agglomeration and mobility restrictions that impede late-stage diffusion and completion of crosslinking.

In contrast, silane-functionalized ZrO_2_ (ZrO_2_VS) shows a different balance. The UPe/ZrO_2_VS(c) formulation (5 wt%) exhibits the longest early-stage time among the modified systems (t_50_ = 19 min), attributable to viscosity increase and diffusion constraints at the start of cure [36]. However, the modified series achieves higher α_90_ and α_final_, consistent with improved dispersion and the presence of interfacial vinyl groups from VTES that remain available for reaction at later times. Overall, the results support a complex interplay between filler content, particle geometry/dispersion, and surface chemistry in governing cure kinetics [35].

### 3.2. Morphological Characteristics and Particle Size Analysis of Unmodified and VTES-Coated ZrO_2_ Nanoparticles and Flax Fabric

**Figure 5:**
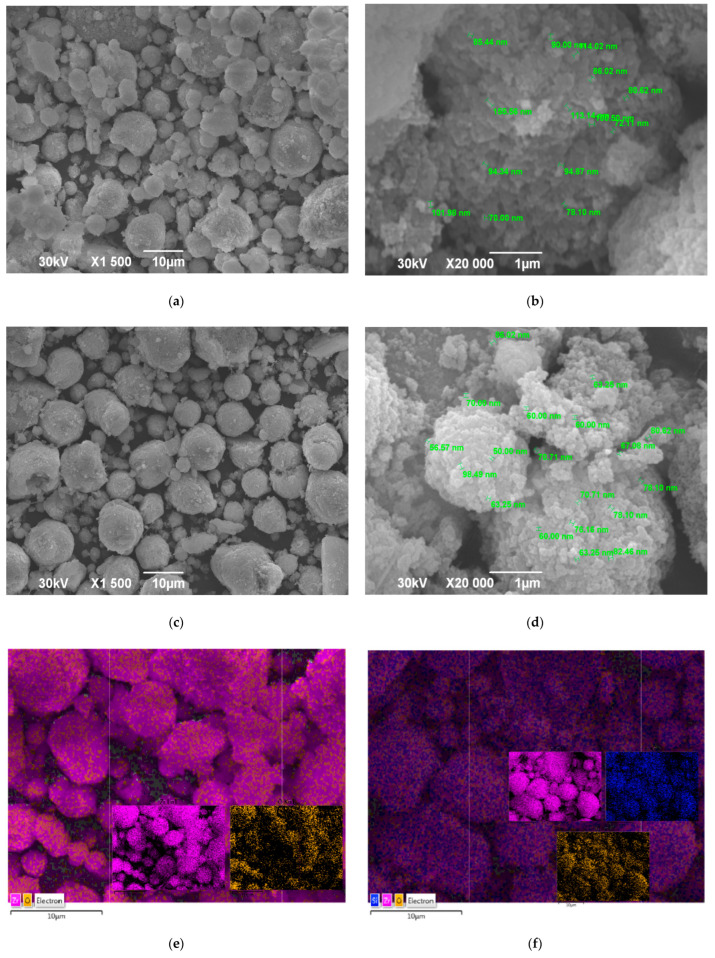
SEM micrographs of (**a**,**b**) umodified ZrO_2_ nanoparticles, (**c**,**d**) VTES-coated n-ZrO_2_, and (**e**,**f**) EDS analysis used for the production of composite laminate materials.

**Figure 6:**
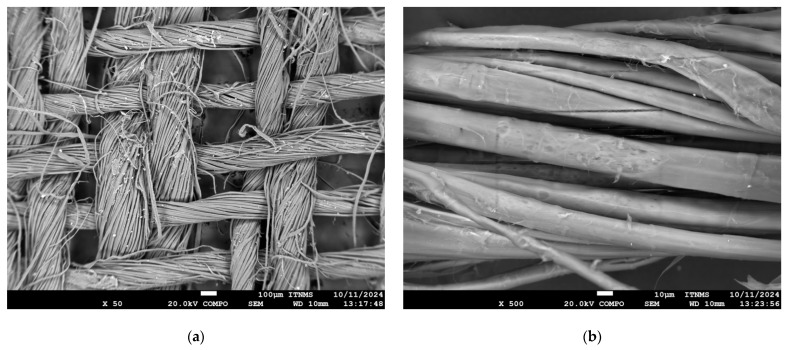
SEM micrographs of flax fabric at (**a**) 50× and (**b**) 500× magnification.

The morphological characteristics of ZrO_2_ nanoparticles and flax fabric used to produce composite laminates were obtained from SEM micrographs (Figure 5 and Figure 6). SEM micrographs of unmodified and VTES-coated ZrO_2_ nanoparticles (magnification of 1500 and 20,000) are shown in Figure 5. Pristine ZrO_2_ particles are 80 nm in size and spherical in shape, but SEM micrographs reveal a significant degree of agglomeration [37], with clusters reaching up to 10 µm, which is in agreement with the results obtained by determining the particle size distribution (Table S1, Figure S2). This agglomeration is likely due to van der Waals forces and the high surface energy of unmodified nanoparticles, which can hinder their uniform dispersion in polymer matrices. Such behavior underlines the necessity for surface modification (e.g., silanization) to enhance compatibility and dispersion within the polymer matrix [18]. Such chemical treatment would alter the surface charge of the nanoparticles, thereby reducing interparticle interactions and improving dispersion. EDS analysis confirm successful surface modification of n-ZrO_2_ with VTES coupling agent (Figure 5e,f). A significant present and uniform dispersion of silicon (Si) were presented (Figure 5f).

It was determined by SEM microscopy that the flax fibers are twisted at an angle of 0/90° into the fabric designated Twill 2/2 weave (Figure 6). This type of weaving should provide optimal mechanical properties of the final composite material. A balanced weave structure is known to provide good mechanical stability and uniform load distribution in composite laminates. The flax fiber surfaces appear slightly rough, which may be beneficial for fiber–matrix adhesion, as the surface texture can increase the mechanical interlocking at the interface. However, further surface treatments could be considered to enhance chemical bonding and reduce moisture sensitivity, both common concerns in natural fiber-reinforced composites.

Overall, the SEM observations support the importance of nanoparticle surface treatment and confirm the structural integrity and suitability of the flax fabric architecture for composite reinforcement applications.

### 3.3. Mechanical Characteristics

**Figure 7:**
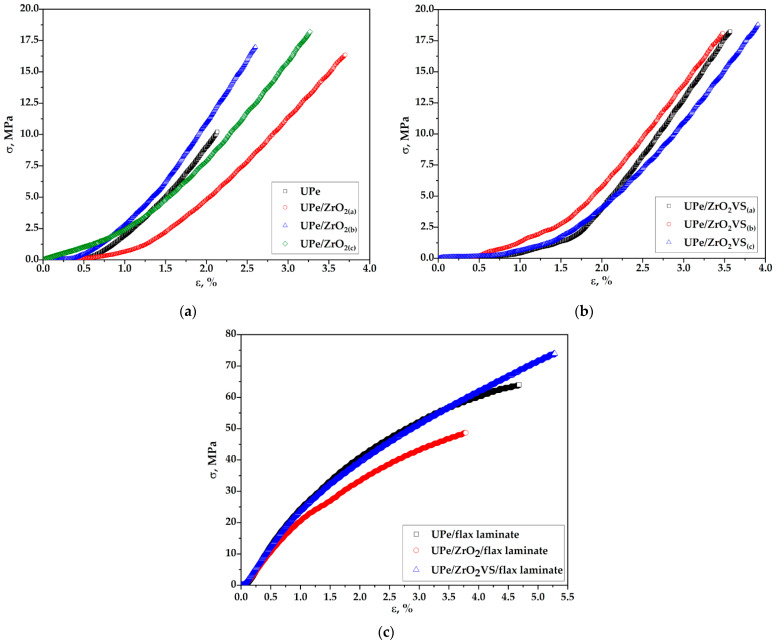
The stress-strain curves of the cured (**a**) UPe, (**b**) UPe/ZrO_2_(n) and (**c**) UPe/ZrO_2_VS(n) nanocomposites.

A significant improvement in the mechanical properties of UPe resin was achieved by incorporating VTES-modified n-ZrO_2_ nanoparticles. To investigate the influence of the structure of vinyl functional groups of n-ZrO_2_ on the tensile properties of nanocomposite materials based on UPe resin, the values of the tensile strength (σ_b_), unit elongation (ε_b_), and modulus of elasticity (E) were determined by uniaxial tensile tests. The results of testing tensile properties of nanocomposites are shown in Table 3, while the corresponding curves showing the dependence of tensile strength on unit elongation are shown in Figure 7.

Table 3 shows that incorporation of unmodified ZrO_2_ nanoparticles into the UPe polymer matrix led to an improvement in its tensile properties. An addition of 1.5 wt.% of pristine filler particles provides a 53.5% increase in tensile strength (σ_b_) compared to neat UPe resin. Further incorporation of powdered reinforcement, 3.0 wt.% and 5.0 wt.%, leads to obtaining more improvement of tensile strength compared to pure UPe resin, 58.6% and 76.8%, respectively. There is an inconsistency in the elastic behavior of corresponding composites since the addition of 1.5 wt.% and 5.0 wt.% of fillers makes the final product more flexible, while the presence of 3.0 wt.% stiffens it. Such a phenomenon can be attributed to the sample manufacturing process, i.e., filler particle distribution within the polymer matrix.

Elastic modulus (*E*) significantly increases with rising concentrations of unmodified ZrO_2_ nanoparticles, according to the remarked tensile strength (up to 27.5% for UPe/ZrO_2_(b) composite). Introduction of reactive vinyl groups on the ZrO_2_ nanoparticles’ surface results in a substantial enhancement in σ_b_ and ε_b_. In this case, the obtained results show an increase in tensile strength that is comparable with the addition of the highest amount of unmodified ZrO_2_ nanoparticles. In addition, there is no significant difference in σ_b_ regarding the amount of modified filler particles. Specifically, the addition of 5.0 wt.% VTES-functionalized ZrO_2_ nanoparticles led to a 76.1% increase in tensile strength, while for the UPe/ZrO_2_VS(b) composites is 69.1%, compared to pure UPe resin.

UPe resin, reinforced with flax fiber, demonstrates significantly higher tensile strength than resin filled only with powdered ZrO_2_. This improvement is attributed to the higher volume fraction of reinforcing flax fabric, which serves as the primary load-bearing component of the composite. A notable enhancement in the mechanical properties of the final flax fiber-reinforced composite indicates a synergistic effect between the nanoparticles and the fabric reinforcement in this configuration.

Flax fiber-based composites using UPe as a matrix represent a sustainable and technically viable alternative to synthetic fiber-reinforced composites, aligning with the global shift toward bio-based materials [11]. Recent studies confirmed that the main challenge is improving fiber-matrix compatibility in thermosetting composites [4,38]. Inorganic fillers such as metal oxides (e.g., Al_2_O_3_, ZrO_2_, MgO) and ceramic nanoparticles (e.g., silica) have been increasingly used to enhance the performance of fiber/UPe composites [4,38]. These fillers improve mechanical strength, thermal stability, and resistance to moisture. Studies have shown that well-dispersed nanoparticles improve interfacial bonding and load transfer within the matrix, especially when combined with surface-treated flax fibers. The hybridization of flax fibers with inorganic fillers thus presents a promising route toward high-performance, eco-friendly composite materials for structural and semi-structural applications.

### 3.4. Dynamic-Mechanical Characteristics

**Figure 8:**
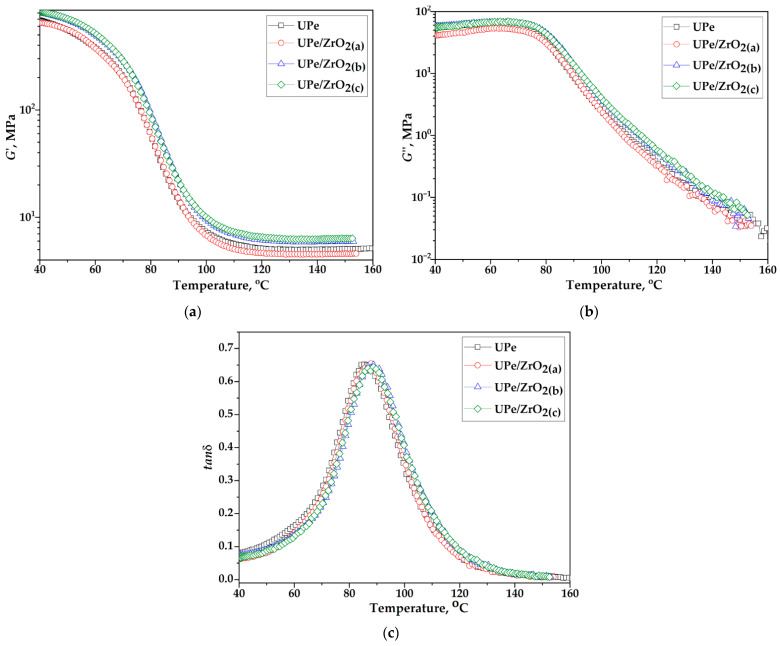
(**a**) Storage modulus, (**b**) loss modulus, and (**c**) damping factor dependence with temperature of UPe/ZrO_2_ composites.

**Figure 9:**
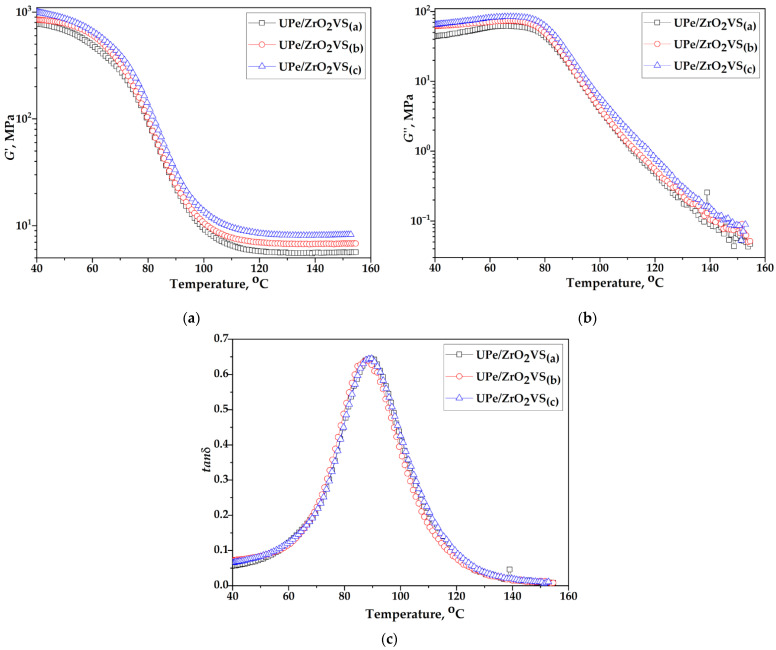
(**a**) Storage modulus, (**b**) loss modulus, and (**c**) damping factor dependence with temperature of UPe/ZrO_2_VS composites.

The chemical composition and macromolecular structure of polymers, along with their interactions with nanofillers, significantly influence the crystallinity and dynamic-mechanical behavior of composite materials. Dynamic mechanical analysis (DMA) of the composite provided key parameters as a function of temperature, time, and frequency: storage modulus (*G*′), loss modulus (*G*″), damping factor (tan (δ)), and glass transition temperature (*T_g_*). Polymers exhibit viscoelastic behavior, combining both elastic and viscous responses. In this context, the storage modulus (*G*′) reflects the material’s elastic (energy-storing) component, while the loss modulus (*G*″) represents its viscous (energy-dissipating) behavior. The damping factor, defined as the ratio tan (δ) = G″/G′, quantifies the extent of viscoelasticity, providing insight into molecular mobility and phase transitions within the material. The response of composite materials to a linear increase in temperature from 40 °C to 160 °C at a constant frequency of 1 Hz was monitored. Figure 8 and Figure 9 show DMA diagrams of crosslinked UPe resin, nanocomposite, and corresponding laminates reinforced with unmodified and ZrO_2_VS, while Table 4 shows the values of *G*’, *G*”, tan (δ) peak height, and *T*_g_.

Adding ZrO_2_ nanoparticles increases the storage modulus (*G*’_GS_) in the glassy region (T < 50 °C), compared to pure resin relative to neat UPe. The largest gain among the unmodified systems occurs at 5.0 wt% n-ZrO_2_, giving +30.9% versus the pure resin. Silane modification further amplifies this effect: the 5.0 wt% ZrO_2_VS nanocomposite shows +60.8% versus neat UPe and +22.9% relative to the unmodified 5.0 wt% counterpart (Table 4). These increases are consistent with a stiffer interphase and improved stress transfer arising from better particle–matrix coupling and reduced chain mobility near the filler surface [2].

In the rubbery region, the same loading dependence is observed, with the highest *G*’_RP_ value again for the UPe/ZrO_2_VS (5.0 wt%) nanocomposite. Across the entire temperature range examined, the loss modulus (G″) remains below G′, indicating a predominantly elastic response of the cured materials.

The temperature dependence of tan(δ) shows the presence of a peak in the range from 85 to 89 °C, where the temperature at which this peak occurs represents the transition temperature to the glassy state of the tested samples. Based on the tan(δ) curves (Table 4, Figure 8 and Figure 9), it can be seen that the presence of unmodified and VTES-modified n-ZrO_2_ nanoparticles did not lead to significant changes in the glass transition temperature of the polymer matrix.

*T*_g_ is a good parameter for monitoring the curing process of a thermosetting material, which increases considerably regarding the changes in chemical conversion during its solidification [39]. Considering thermosets, there is a linear increase in the degree of conversion in the initial stage of curing, which becomes exponential in the final stage when the process is diffusion-controlled as the polymer is completely solidified [40]. By reaching a high degree of conversion, when the reaction rate is low, measurement the actual value of *T*_g_ is sensitive and becomes difficult. In other words, the technique employed such as FTIR is not suitable for detecting small changes in chemical conversion, markedly in the final stages of the curing process [40]. Monitoring the curing process by FTIR (Table 2) shows that unmodified ZrO_2_ nanocomposites reach final conversions α_final_ ≈ 85–86% at medium loadings, but drop to about 78% at the highest loading, whereas VTES-functionalized ZrO_2_ systems reach higher final conversions of about 90–95%. Consistent with this, DMA (Table 4) reveals only modest *T*_g_ shifts from 85.4 °C for neat UPe to ≈87–89.5 °C for UPe/ZrO_2_ and UPe/ZrO_2_VS nanocomposites. Such results indicate that the presence of pristine filler nanoparticles sterically hinders the successful orientation of the functional segments from polymer matrix and curing agents making the final material incompletely solidified. In contrast, modified ZrO_2_ nanoparticles act as anchoring spots between mainly reactive phases within the composite, improving the cross-link density and consequently providing a higher degree of conversion. However, these differences in degree of conversion are potentially smaller due to the technique used, since the *T*_g_ values are negligibly changed.

**Figure 10:**
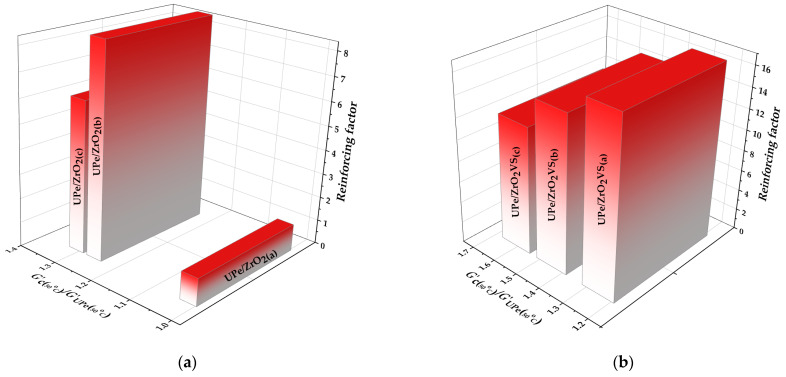
Ratio of *G*’_GS50 °C_ of (**a**) UPe/ZrO_2_ and (**b**) UPe/ZrO_2_VS composites and pure UPe matrix with calculated reinforcement factor at 50 °C.

Calculation of the reinforcing factor was used to measure the effect of ZrO_2_ and ZrO_2_VS particles on the mechanical properties of the UPe polymer matrix. Figure 10 shows the reinforcement factor of unmodified and VTES-modified n-ZrO_2_ particles in relation to pure crosslinked UPe resin. Based on the calculated values, it can be observed that both unmodified and VTES-modified n-ZrO_2_ particles showed a positive reinforcing factor on the UPe properties. As a reinforcing factor that depends on shape, size, and interfacial interaction between filler and polymer matrix, we can confirm that a higher reinforcing effect was achieved in the case when VTES-modified n-ZrO_2_ particles were used (from 12.2 to 16.9). The obtained results indicate that better compatibility and interfacial adhesion are accomplished between the non-polar UPe matrix and polar flax fabric when the UPe matrix is pre-filled using ZrO_2_VS coated nanoparticles. The lowest reinforcing effect was observed in the UPe/ZrO_2_ sample when n-ZrO_2_ particles were added in an amount of 1.0 wt.%. The addition of unmodified n-ZrO_2_ particles positively influences the UPe properties, but the effect is ≈15.4 times lower compared to the sample with the same amount of modified n-ZrO_2_ particles (calculated for UPe/ZrO_2_(a) and UPe/ZrO_2_VS(a)). These trends provide an indication that silane functionalization significantly enhances interfacial adhesion and load transfer efficiency in the studied systems. The lower reinforcing effect of the unmodified n-ZrO_2_ particles can be attributed to the higher tendency for particle agglomeration and lower interfacial adhesion.

**Figure 11:**
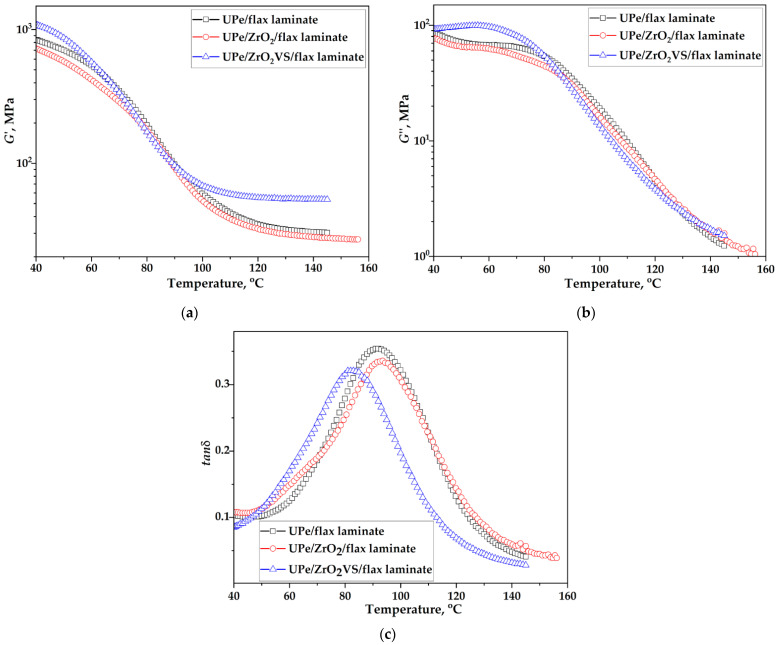
(**a**) Storage modulus, (**b**) loss modulus, and (**c**) damping factor dependence with temperature of UPe/ZrO_2_VS laminates.

Figure 11 illustrates that the flax-reinforced composite exhibits significantly higher storage modulus (G′) values at elevated temperatures compared to composites containing only nanoparticle fillers. The flax-reinforced composite, containing modified filler nanoparticles, displays the highest value of G′ over the entire temperature range, indicating the establishment of a stiffer structure in which the mobility of the macromolecular segments of the polymer matrix is substantially restricted. Therefore, greater cooperation of the macromolecule segments is required to initiate the relaxation process [3]. A similar trend was observed for the loss modulus (*G*″), which additionally confirms enhanced stiffness and energy dissipation capacity in the flax-containing composite. The lower tan(δ) values for the flax-reinforced composites indicate their high elasticity compared to those without incorporated fabric within the polymer matrix. The remarked phenomenon is particularly emphasized in composites containing flax fabric combined with modified filler nano-particles, and it is associated with an increase in matrix/reinforcement adhesion [2]. As a result of these structural characteristics, the *T_g_* of the flax-reinforced composite is higher than that of the nanoparticle-filled counterparts. However, the increase in *T_g_* was relatively modest, as the viscoelastic behavior of the composites is predominantly governed by the polymer matrix. This is evident from the comparable *T_g_* values across nanocomposites with varying nanoparticle loadings (Table 4).

These results indicate that incorporation of flax cloth mainly stiffens the interfacial area and improves load transfer, while the overall crosslink density (and thus *T*_g_) remains only slightly altered. The relatively small *T*_g_ changes, despite the large gains in storage modulus, indicate that the global crosslink density of the polyester network is only slightly modified. The dominant mechanism is instead attributed to the stiffening of the interfacial area and locally restricted chain mobility in the vicinity of the silane-functionalized ZrO_2_ and flax fibers, which improves stiffness and damping behavior without a major shift in bulk *T*_g_.

### 3.5. Tensile Fracture Surface Observation

**Figure 12:**
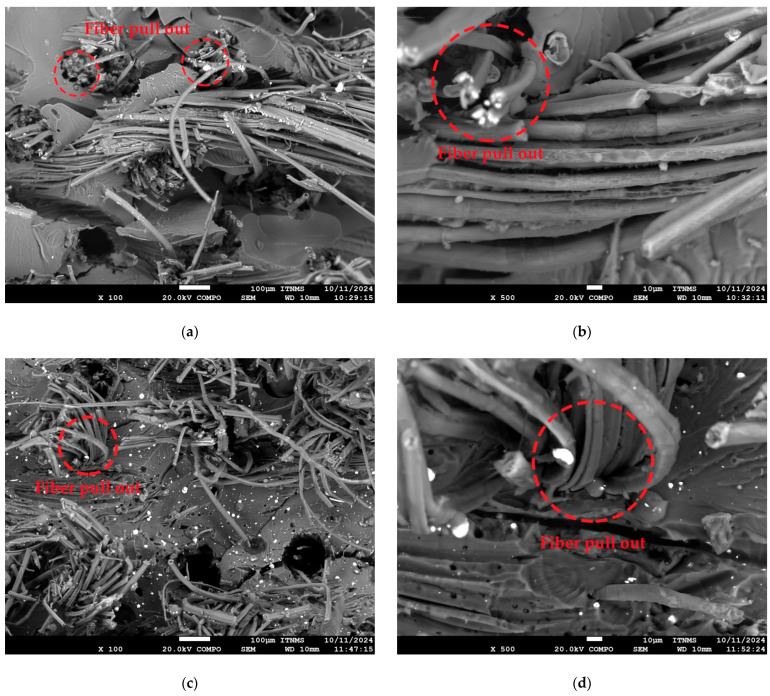

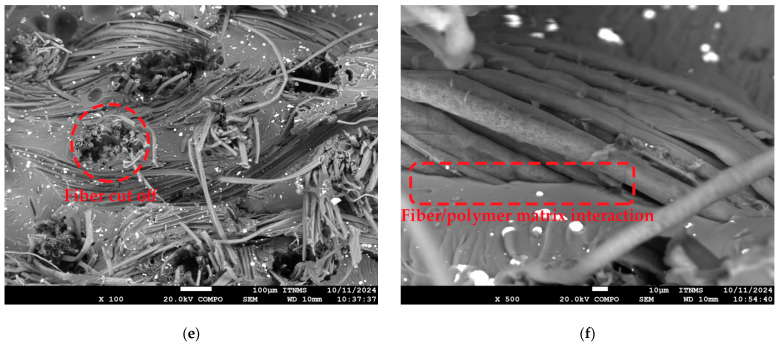
SEM analysis of the tensile fracture surfaces of the (**a**,**b**) UPe/flax laminate, (**c**,**d**) UPe/ZrO_2_/flax laminate and, (**e**,**f**) UPe/ZrO_2_VS/flax laminate.

Morphological observation of the tensile fracture surface of the laminates provides insight into bonding between fibers and polymer matrix [41]. The fiber pull-out zone of UPe/flax fiber/ZrO_2_ laminate differs depending on the interphase interactions/bonding between fibers and UPe resin. The SEM analysis of the tensile fracture surfaces offers valuable insight into the fiber-matrix interfacial behavior in the UPe/flax fiber/ZrO_2_ laminates. As shown in Figure 12, the morphology of the fiber pull-out zones varies depending on the presence and type of ZrO_2_ nanoparticles in the resin. When ZrO_2_ nanoparticles, especially those modified with vinyl functional groups, are incorporated into the matrix, the bonding between flax fibers and the UPe resin appears significantly improved. Fibers’ periphery is properly embodied in the UPe matrix, especially in a laminate which contains vinyl-modified ZrO_2_ nanoparticles.

The images reveal that the flax fibers are more thoroughly embedded within the matrix, indicating effective wetting and mechanical interlocking. In particular, the laminate containing vinyl-modified ZrO_2_ shows a more cohesive interphase, where the matrix tightly adheres to the fiber surface. This suggests that the vinyl groups facilitate dual interfacial interactions: one between the nanoparticles and the polymer matrix, and another between the nanoparticles and the natural fiber surface. As a result, fiber pull-out is reduced, and the fracture surface exhibits a more ductile failure mode, indicating improved energy absorption and better stress transfer across the interface.

**Figure 13:**
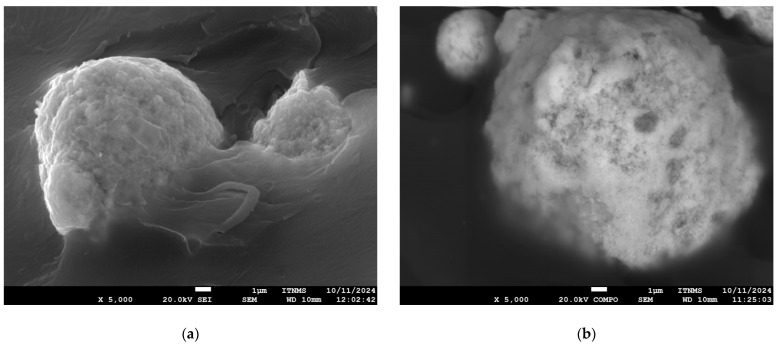
Interfacial bonding of (**a**) UPe and (**b**) ZrO_2_VS particles.

These findings support the conclusion that surface-modified nanoparticles not only improve nanoparticle dispersion but also act as interfacial bridges, enhancing the compatibility between hydrophilic flax fibers and the hydrophobic UPe matrix as it shown in Figure 13.

## 4. Conclusions

This study demonstrated the development of a sustainable and high-performance flax fiber-reinforced composite by integrating zirconium oxide (ZrO_2_) nanoparticles into a styrene-free unsaturated polyester (UPe) matrix. The incorporation of both unmodified and surface-functionalized (VTES-coated) ZrO_2_ nanoparticles significantly influenced the interfacial bonding, mechanical strength, and thermal stability of the resulting composite laminates.

In summary, VTES-functionalized ZrO_2_ nanoparticles enhance interfacial efficiency and mechanical performance in styrene-reduced UPe without penalizing cure. FTIR shows comparable or slightly higher late-stage conversion for UPe/ZrO_2_VS compared to UPe/ZrO_2_ (90–95% and 78–86%, respectively). DMA indicates clear gains in storage modulus in both glassy and rubbery regions, with ZrO_2_VS outperforming unmodified ZrO_2_ (increases from 540.7 MPa (UPe) to 680–870 MPa for the nanocomposites). In flax-reinforced laminates, this translates into the highest storage modulus over the whole temperature range (up to 866 MPa), reduced tan(δ), and improved tensile strength with retained ductility, consistent with strengthened matrix–reinforcement adhesion. Tg shifts remain modest (from 85.4 °C for neat UPe to ≈87–89.5 °C for UPe/ZrO_2_ and UPe/ZrO_2_VS), and from 91.6 °C to 93.5 °C when moving from the flax laminate without nanoparticles to the ZrO_2_VS/flax laminate, suggesting that viscoelastic behavior is still largely governed by the UPe matrix and that interphase stiffening dominates the response. Processing remains compatible with VARTM, ensuring good laminate consolidation.

Overall, combining a styrene-reduced UPe matrix with natural flax fibers and silane-modified ZrO_2_ offers a practical route to sustainable composites with enhanced thermomechanical and tensile performance, positioning flax/ZrO_2_/UPe systems as promising green alternatives to conventional synthetic fiber-reinforced composites.

## Figures and Tables

**Table 1:** FlaxdryTM BL200 product datasheet.

Product Description			
Nature of product	Twill 2-2 fabric		
Type of yarn	Warp: Half wet/Weft: Half wet		
Linear density	Warp: 9.5 Nm/Weft: 9.5 Nm		
Flax origin	100% European Flax		
Weaving style	Warp: 10.2 yarns/cm/Warp: 10.1 yarns/cm		
Weight distribution	Warp: 49.8%/Warp: 50.2%		
Property			
Moisture content	9 ± 1%		NF EN ISO 3344 [24]
Surface weight	220 ± 22 g m^−2^		NF G07-150 [25]
Fiber density	1.45 g cm^−3^		
Tensile properties ^a^			
	Weft direction	Warp direction	Standard
Tensile strength	58 ± 2 MPa	61 ± 6 MPa	ISO 527-5 [26]
Young Modulus ^b^	6 ± 1 GPa	7 ± 1 GPa	ISO 527-5 [26]
Elongation at break	1.8 ± 0.1%	1.5 ± 0.2%	ISO 527-5 [26]
Thickness of 1 impregnated ply	0.59 ± 0.02 mm	0.59 ± 0.02 mm	NF EN ISO 5084 [27]

^a^ Flax/Bio-epoxy composite at 29 ± 1% of fabric volumetric fraction made by vacuum infusion at 0.4 mBar; ^b^ Modulus measured between 0.02 and 0.1% deformation.

**Table 2:** Times needed to achieve 50% (α_50%_) and 90% (α_90%_) and final conversion (α_final_) of vinyl toluene.

Sample	t (α_50%_), min	t (α_90%_), min	α_final_, %
UPe/ZrO_2_(a)	25.0	-	85.0
UPe/ZrO_2_(b)	12.0	-	86.0
UPe/ZrO_2_(c)	19.0	-	78.0
UPe/ZrO_2_VS(a)	12.5	80.0	95.0
UPe/ZrO_2_VS(b)	7.5	120.0	90.0
UPe/ZrO_2_VS(c)	19.0	61.0	95.0

**Table 3:** Stress at break (*σ*_b_), elongation at break (*ε*_b_), and tensile modulus (*E*) of cured UPe and UPe/ZrO_2_(n) nanocomposites.

Sample	*σ*_b_, MPa	*ε*_b_, %	*E*, MPa
UPe	10.71 ± 0.07	2.27 ± 0.19	479.86 ± 10.95
UPe/ZrO_2_(a)	16.44 ± 0.12	3.33 ± 0.53	495.73 ± 77.79
UPe/ZrO_2_(b)	16.99 ± 0.09	2.18 ± 0.17	611.98 ± 50.07
UPe/ZrO_2_(c)	18.94 ± 0.11	3.27 ± 0.22	499.18 ± 25.12
UPe/ZrO_2_VS(a)	18.25 ± 0.15	3.57 ± 0.09	500.20 ± 50.20
UPe/ZrO_2_VS(b)	18.11 ± 0.11	3.48 ± 0.15	458.38 ± 41.10
UPe/ZrO_2_VS(c)	18.86 ± 0.09	3.91 ± 0.11	455.76 ± 37.26
UPe/flax laminate	50.87 ± 3.07	3.84 ± 0.08	2384.9 ± 85.96
UPe/ZrO_2_/flax laminate	58.27 ± 6.08	3.42 ± 0.19	1735.9 ± 128.31
UPe/ZrO_2_VS/flax laminate	72.18 ± 3.62	5.29 ± 0.38	1579.05 ± 41.81

**Table 4:** DMA results of UPe and corresponding composites and laminates.

Sample	*G′*_GS50C_, MPa	*G*′_RP130C_, MPa	*T*_g_ (tan(δ)), °C	Tan(δ) Peak Height
UPe	540.69	5.02	85.36	0.65
UPe/ZrO_2_(a)	549.77	4.53	87.76	0.66
UPe/ZrO_2_(b)	680.78	5.92	88.76	0.65
UPe/ZrO_2_(c)	707.51	6.26	88.71	0.64
UPe/ZrO_2_VS(a)	677.75	5.58	89.52	0.65
UPe/ZrO_2_VS(b)	783.30	6.77	87.66	0.64
UPe/ZrO_2_VS(c)	869.54	8.15	88.64	0.65
UPe/flax laminate	701.86	32.11	91.56	0.35
UPe/ZrO_2_/flax laminate	575.91	29.62	82.05	0.32
UPe/ZrO_2_VS/flax laminate	866.27	54.26	93.48	0.34

## Data Availability

The original contributions presented in this study are included in the article/Supplementary Material. Further inquiries can be directed to the corresponding author.

## Supplementary Materials

The following supporting information can be downloaded at: https://www.mdpi.com/article/10.3390/polym18010070/s1, Figure S1: ZrO_2_ nanoparticles surface treatment with VTES; Figure S2: Particle distribution obtained by the volumetric method; Figure S3. (a) Time changing of height of the reactive C=C out-of-plane deformation peak at 909 cm^−1^, (b) Constant reference ester C=O peak height around 1734 cm^−1^ (representative example for UPe/ZrO_2_(b)); Figure S4. (a) The degree of the solvent conversion (αt as a function of curing time), (b) Curing rate vs. time plot; Table S1. Size of n-ZrO_2_ particles determined on the analyzer.

## References

[1] Carvalho J.P.R.G., Lopes F.P.D., Simonassi N.T., Neves A.C.C., de Carvalho E.A., Monteiro S.N., Vieira C.M.F. (2022). Methodological Analysis of Composites Green Polyurethane Resin Reinforced with Jute Fabric. Case Stud. Constr. Mater..

[2] Hamdan M.H.M., Siregar J.P., Ahmad M.R., Asghar A., Tezara C., Jamiluddin J., Zalinawati M. (2021). Characterisation of the Woven Fabric of Jute, Ramie and Roselle for Reinforcement Material for Polymer Composite. Mater. Today Proc..

[3] Shah D.U., Schubel P.J., Clifford M.J., Licence P. (2013). Fatigue Life Evaluation of Aligned Plant Fibre Composites through S-N Curves and Constant-Life Diagrams. Compos. Sci. Technol..

[4] Velmurugan G., Siva Shankar V., Kalil Rahiman M., Prathiba R., Dhilipnithish L.R., Khan F.A. (2023). Effectiveness of Silica Addition on the Mechanical Properties of Jute/Polyester Based Natural Composite. Mater. Today Proc..

[5] Vázquez-Núñez E., Avecilla-Ramírez A.M., Vergara-Porras B., López-Cuellar M.R. (2021). Green composites and their contribution toward sustainability: A review. Polym. Polym. Composites..

[6] Ahmed M.S., Haque M., Khanand R.A., Miah A.H. (2024). A Review on Properties of Natural Fiber Reinforced Polymer Composites: Effect of Gamma Radiation and Nano particles. Int. J. Eng. Mater. Manuf..

[7] Karthika M.R., Deb A., Venkatesh G.S. (2021). An Investigation into Mechanical Properties of Jute–Polyester Laminates for Numerical Prediction of Strength and Failure. Polym. Compos..

[8] More A.P. (2022). Flax Fiber–Based Polymer Composites: A Review. Adv. Compos. Hybrid Mater..

[9] Langhorst A., Zhang D., Berman J., Biraku X., Rieland J., Yu M., Love B., Banu M., Taub A. (2024). Mechanical Property Enhancement of Flax Fibers via Supercritical Fluid Treatment. Sci. Rep..

[10] Li H., Tang R., Dai J., Wang Z., Meng S., Zhang X., Cheng F. (2022). Recent Progress in Flax Fiber-Based Functional Composites. Adv. Fiber Mater..

[11] Yan L., Chouw N., Jayaraman K. (2014). Flax Fibre and Its Composites—A Review. Compos. B Eng..

[12] Saha A.K., Das S., Bhatta D., Mitra B.C. (1999). Study of Jute Fiber Reinforced Polyester Composites by Dynamic Mechanical Analysis. J. Appl. Polym. Sci..

[13] Senthilrajan S., Venkateshwaran N., Naresh K., Velmurugan R., Gupta N.K. (2022). Effects of Jute Fiber Length and Weight Percentage on Quasi-Static Flexural and Dynamic Mechanical Properties of Jute/Polyester Composites for Thin-Walled Structure Applications. Thin-Walled Struct..

[14] Ahmed K.S., Vijayarangan S. (2007). Experimental Characterization of Woven Jute-Fabric-Reinforced Isothalic Polyester Composites. J. Appl. Polym. Sci..

[15] Alia A., Fantozzi G., Godin N., Adrien J., Osmani H., Reynaud P. (2023). Multi-Instrumented Analysis of Fatigue Behavior and Damage Mechanisms in Jute Fiber-Reinforced Polyester Composites. Int. J. Fatigue.

[16] Liu W., Xie T., Qiu R. (2015). Styrene-Free Unsaturated Polyesters for Hemp Fibre Composites. Compos. Sci. Technol..

[17] Noh Y., Odimayomi T., Mahboobeh S., Sendesi T., Youngblood J.P., Whelton A.J. (2022). Environmental and human health risks of plastic composites can be reduced by optimizing manufacturing conditions. J. Clean. Prod..

[18] Rusmirović J.D., Trifković K.T., Bugarski B., Pavlović V.B., Džunuzović J., Tomić M., Marinković A.D. (2016). High Performance Unsaturated Polyester Based Nanocomposites: Effect of Vinyl Modified Nanosilica on Mechanical Properties. Express Polym. Lett..

[19] Patel V.K., Dhanola A. (2016). Influence of CaCO_3_, Al_2_O_3_, and TiO_2_ Microfillers on Physico-Mechanical Properties of Luffa Cylindrica/Polyester Composites. Eng. Sci. Technol. Int. J..

[20] Biswas B., Chabri S., Sawai P., Mitra B.C., Das K., Sinha A. (2018). Effect of Copper Incorporation on the Mechanical and Thermal Behavior of Jute Fiber Reinforced Unsaturated Polyester Composites. Polym. Compos..

[21] Ahmed K.S., Vijayarangan S. (2008). Tensile, Flexural and Interlaminar Shear Properties of Woven Jute and Jute-Glass Fabric Reinforced Polyester Composites. J. Mater. Process Technol..

[22] Alam M.A., Samad U.A., Anis A., Sherif E.-S.M., Abdo H.S., Al-Zahrani S.M. (2023). The Effect of Zirconia Nanoparticles on Thermal, Mechanical, and Corrosion Behavior of Nanocomposite Epoxy Coatings on Steel Substrates. Materials.

[23] Ajith A., Xian G., Li H., Sherief Z., Thomas S. (2015). Surface grafting of flax fibres with hydrous zirconia nanoparticles and the effects on the tensile and bonding properties. J. Compos. Mater..

[24] (1997). Reinforcement Yarns—Determination of Moisture Content.

[25] (1984). Textiles. Tests for Fabrics. Method for the Determination of the Surface Density.

[26] (2021). Plastics—Determination of Tensile Properties—Part 5: Test Conditions for Unidirectional Fibre-Reinforced Plastic Composites.

[27] (1996). Textiles. Determination of Thickness of Textiles and Textile Products.

[28] Song S.Y., Park M.S., Lee J.W., Yun J.S. (2018). Improvement of Dispersion Stability and 3D-Printing Characteristics of Ceramics in Photopolymers by Controlling the Coating Thickness of Silane Coupling Agents. Mater. Chem. Phys..

[29] Ju Y., Ha J., Song Y., Yun J.S., Lee D. (2020). Optimizing the Printability and Dispersibility of Functionalized Zirconium Oxide/Acrylate Composites with Various Nano-to Micro-Particle Ratios. Ceram. Int..

[30] Mijatov S., Vuksanović M.M., Knežević N., Anđelković B., Cvijetić I., Milošević M., Mladenović I.O., Marinković A. (2025). Mechanical Properties of the Bio-Composites: Effect of Kraft Lignin and Flax Fabric to Camphoric Acid Based Unsaturated Polyester Resin’s Reinforcement. Polym. Compos..

[31] (2009). Standard Test Method for Tensile Properties of Thin Plastic Sheeting.

[32] Jyoti J., Singh B.P., Arya A.K., Dhakate S.R. (2016). Dynamic Mechanical Properties of Multiwall Carbon Nanotube Reinforced ABS Composites and Their Correlation with Entanglement Density, Adhesion, Reinforcement and C Factor. RSC Adv..

[33] Singh A.K., Nakate U.T. (2014). Microwave Synthesis, Characterization, and Photoluminescence Properties of Nanocrystalline Zirconia. Sci. World J..

[34] Issa A.A., Luyt A.S. (2019). Kinetics of Alkoxysilanes and Organoalkoxysilanes Polymerization: A Review. Polymers.

[35] Kovačević T., Rusmirović J., Tomić N., Marinović-Cincović M., Kamberović Ž., Tomić M., Marinković A. (2017). New Composites Based on Waste PET and Non-Metallic Fraction from Waste Printed Circuit Boards: Mechanical and Thermal Properties. Compos. B Eng..

[36] Dao P.H., Nguyen T.C., Phung T.L., Nguyen T.D., Nguyen A.H., Vu T.N.L., Vu Q.T., Vu D.H., Tran T.K.N., Thai H. (2021). Assessment of Some Characteristics and Properties of Zirconium Dioxide Nanoparticles Modified with 3-(Trimethoxysilyl) Propyl Methacrylate Silane Coupling Agent. J. Chem..

[37] Ramesh M., Palanikumar K., Reddy K.H. (2017). Plant Fibre Based Bio-Composites: Sustainable and Renewable Green Materials. Renew. Sustain. Energy Rev..

[38] Romanzini D., Lavoratti A., Ornaghi H.L., Amico S.C., Zattera A.J. (2013). Influence of Fiber Content on the Mechanical and Dynamic Mechanical Properties of Glass/Ramie Polymer Composites. Mater. Des..

[39] Bernath A., Kärger L., Henning F. (2016). Accurate Cure Modeling for Isothermal Processing of Fast Curing Epoxy Resins. Polymers.

[40] Zhang Y., Adams R.D., da Silva L.F.M. (2014). Effects of Curing Cycle and Thermal History on the Glass Transition Temperature of Adhesives. J. Adhes..

[41] Adhikari J., Biswas B., Chabri S., Bandyapadhyay N.R., Sawai P., Mitra B.C., Sinha A. (2017). Effect of Functionalized Metal Oxides Addition on the Mechanical, Thermal and Swelling Behaviour of Polyester/Jute Composites. Eng. Sci. Technol. Int. J..

