# Supplementary material for: Green Fiber-Reinforced Laminates: Styrene-Free UPe with VTES-Functionalized ZrO2 and Flax Fabrics
Source: Polymers (Basel). 2025 Dec 26;18(1):70. doi: 10.3390/polym18010070 (PMC12788117; doi:10.3390/polym18010070)
Supplement: Supplementary file 1 [file polymers-18-00070-s001.zip › polymers-4030134-supplementary.pdf]

Green Fiber-Reinforced Laminates: Styrene-Free UPe with VTES-Functionalized ZrO_2 and Flax Fabrics

**Slavko Mijatov [1], Milica Rančić [2], Tihomir Kovačević [1], Jelena Vujančević [3], Vladimir B. Pavlović [4]
and Jelena D. Gržetić [1,*]**

[1] Military Technical Institute, Ratka Resanovića 1, 11030 Belgrade, Serbia;
slavko.mijatov@mod.gov.rs (S.M.);
tkovacevic@tmf.bg.ac.rs (T.K.)

[2] Faculty of Forestry, University of Belgrade, Kneza Višeslava 1, 11030 Belgrade, Serbia;
milica.rancic@sfb.bg.ac.rs

[3] Institute of Technical Sciences of SASA, Knez Mihailova 35/IV, 11000 Belgrade, Serbia;
jelena.vujancevic@itn.sanu.ac.rs

[4] Faculty of Agriculture, University of Belgrade, Nemanjina 6, Zemun, 11080 Belgrade, Serbia;
vladimirbpavlovic@gmail.com

* Correspondence: jrusmirovic@tmf.bg.ac.rs

2.2 Preparation of the VTES-coated ZrO_2 nanoparticles

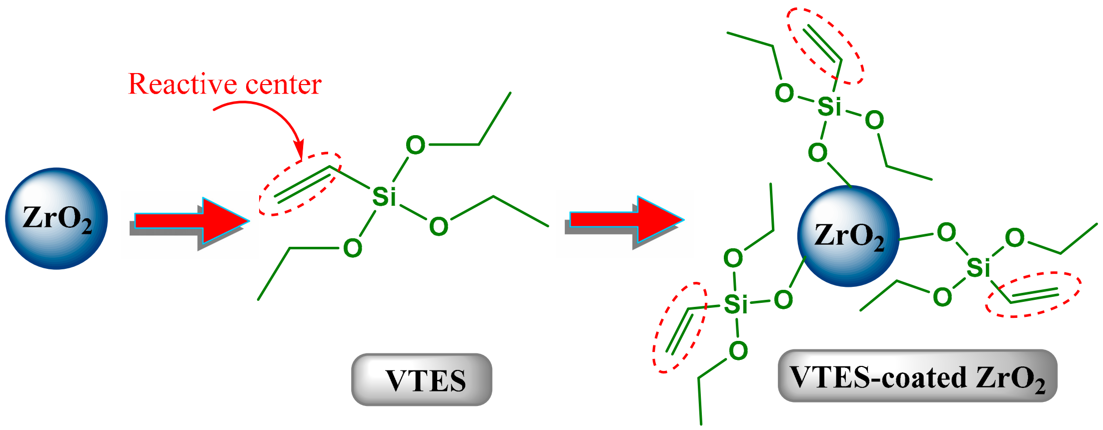

Figure S1. ZrO_2 nanoparticles surface treatment with VTES

3.1. Particle Size Analysis

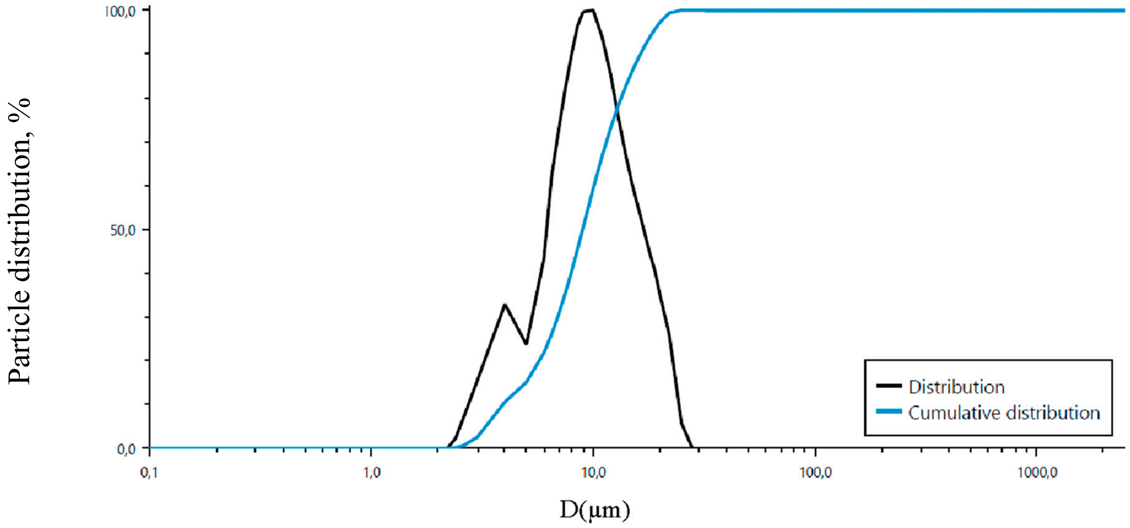

Figure S2. Particle distribution obtained by the volumetric method

Table S1. Size of n-ZrO_2 particles determined on the analyzer

Particle size distribution	D10 [μm]	D50 [μm]	D90 [μm]	Mean value [μm]
n-ZrO_2	3.917	8.967	16.329	10.116

3.1 FTIR analysis and curing kinetic

$$\alpha(\boldsymbol{t}) = \left(\mathbf{1} - \frac{\boldsymbol{A_t}/\boldsymbol{A_{ref}}}{\boldsymbol{A_0}/\boldsymbol{A_{ref}}}\right) \times \mathbf{100} \tag{S1}$$

Where $\boldsymbol{A_t}$ is the height of the reactive C=C out-of-plane deformation peak at 909 cm^{-1} at time, $\boldsymbol{A_{ref}}$ is the height of the reference ester C=O peak around 1734 cm^{-1} , and $\boldsymbol{A_0}$ is the initial height of the reactive peak before curing begins.

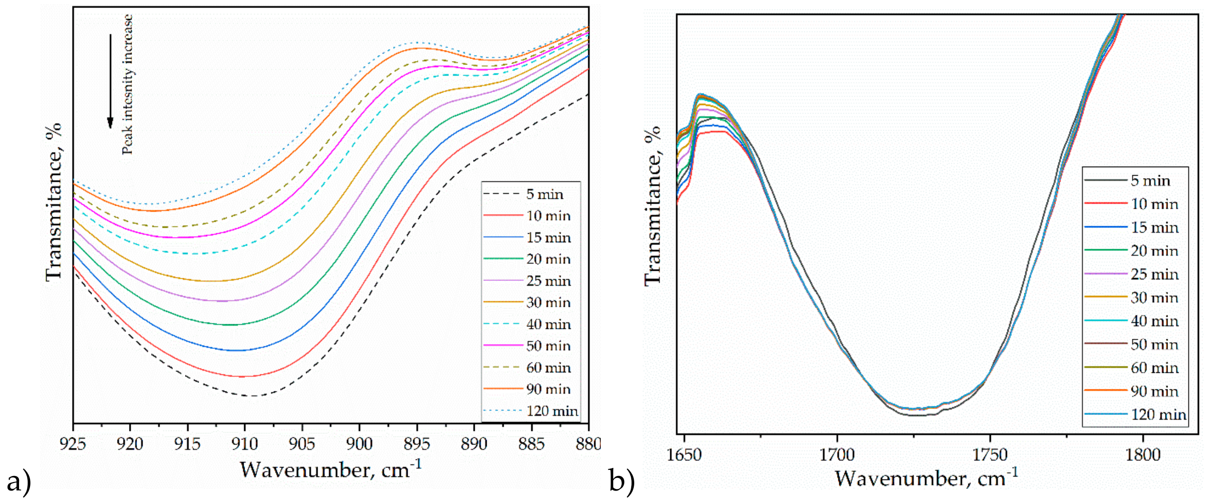

Figure S3. a) Time changing of height of the reactive C=C out-of-plane deformation peak at 909 cm^{-1}, b) Constant reference ester C=O peak height around 1734 cm^{-1} (representative example for UPe/ZrO_2(b))

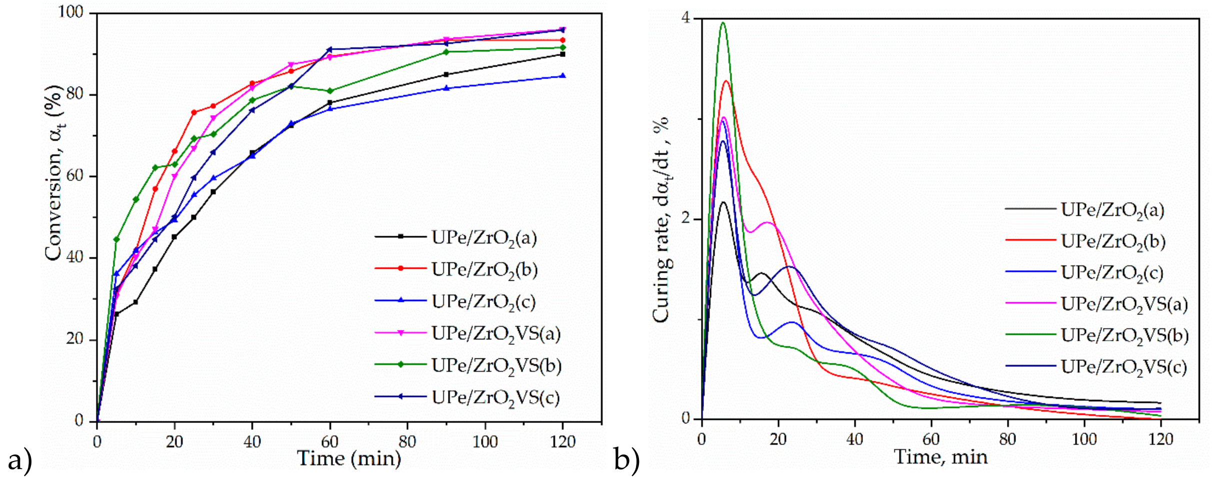

Figure S4. a) The degree of the solvent conversion (α_t as a function of curing time), b) Curing rate vs time plot
